# Microbial Communities and Diversities in Mudflat Sediments Analyzed Using a Modified Metatranscriptomic Method

**DOI:** 10.3389/fmicb.2018.00093

**Published:** 2018-01-31

**Authors:** Yong-Wei Yan, Qiu-Yue Jiang, Jian-Gong Wang, Ting Zhu, Bin Zou, Qiong-Fen Qiu, Zhe-Xue Quan

**Affiliations:** ^1^Key Laboratory for Biodiversity Science and Ecological Engineering, Ministry of Education, Institute of Biodiversity Science, School of Life Sciences, Fudan University, Shanghai, China; ^2^School of Marine Science, Ningbo University, Ningbo, China

**Keywords:** metatranscriptome, SSU rRNA, community composition, intertidal mudflat, bias

## Abstract

Intertidal mudflats are land–sea interaction areas and play important roles in global nutrient cycles. However, a comprehensive understanding of microbial communities in these mudflats remains elusive. In this study, mudflat sediment samples from the Dongtan wetland of Chongming Island, the largest alluvial island in the world, were collected. Using a modified metatranscriptomic method, the depth-wise distributions of potentially active microbial communities were investigated based on small subunit ribosomal RNA (SSU rRNA) sequences. Multiple environmental factors were also measured and analyzed in conjunction with the prokaryotic composition profiles. A prokaryotic diversity analysis based on the metatranscriptome datasets revealed two or threefold higher diversity indices (associated with potentially active microbes participating in biogeochemical processes in Dongtan) compared with the diversity indices based on 16S rRNA gene amplicons. Bacteria were numerically dominant relative to archaea, and the potentially active prokaryotic taxa were mostly assigned to the bacterial phyla *Chloroflexi, Acidobacteria*, and *Bacteroidetes* and the classes *Delta-* and *Gamma-proteobacteria*, along with the archaeal lineages phylum *Bathyarchaeota* and the order *Thermoplasmatales*. The total nitrogen and carbon content of the sediment samples were environmental factors that significantly affected the depth-wise distributions of both bacterial and archaeal communities. Furthermore, the activity of potentially active taxa (including the prevalent order *Desulfobacterales* and family *Anaerolineaceae*) appeared to be significantly underestimated by PCR-based methods, notably at the DNA level, and indicates that using normal PCR amplification of DNA limits the study of potential microbial activity. This is the first study of potentially active microbial communities in depth-wise sediments from Dongtan. The improved knowledge of microbial communities in Dongtan provides a foundation for exploring biogeochemical cycling and microbial functions.

## Introduction

The Dongtan wetland, located at the eastern end of Chongming Island, is an estuarine intertidal wetland of the Yangtze River estuary with a large mudflat on the east beach. Being the largest alluvial island in the world (Gan et al., 2009), Chongming Island has large amounts of organic matter. It was reported that the Yangtze River carries ∼4.8 × 10^8^ tons of sediment to the mudflats annually and about half of the sediment accumulates in this estuary region (Milliman et al., 1985; Song et al., 2013). The nutrients contained in the sediments can be utilized as primary or secondary substrates for the microbial processes, with depth-wise dynamics driving nutrient transformations and energy fluxes. These processes are considered to be of key importance for ecosystem stabilization in intertidal wetlands (Blum and Mills, 2012), and the processes related to carbon (C), nitrogen (N), and sulfur (S) are thought to have significant roles in global biogeochemical cycles because of their increasing influences on greenhouse gas production (Bauer et al., 2013; Zeleke et al., 2013).

Considering the significant eco-functions of Dongtan, studies have been carried out to investigate which microbial communities were involved in the C-, N-, and S-related processes (Zeleke et al., 2013; Zhang et al., 2013; Hu et al., 2014). However, all of these studies involved PCR-dependent amplifications of 16S rRNA genes or functional genes, and the focus was only on several process-related microbe groups, such as methanogenic archaea, sulfate-reducing bacteria, and ammonium-oxidizing microbes. Therefore, more research is needed in order to understand which main microbial groups are present in Dongtan, which activities are performed, and how the depth-wise distributions of the microbes respond to environmental changes. The drawbacks underlying the use of environmental DNA methods include interference from DNA from dead cells and free DNA adsorption into the soil over long periods (Luna et al., 2002; Carvalhais and Schenk, 2013). Thus, PCR-dependent DNA amplification techniques could bias the measurements of physiologically active microbial communities.

As an indicator of potential physiological activity (Blazewicz et al., 2013), studies have used ribosomal RNA (rRNA) to profile potentially active microbes in diverse environments (Lanzén et al., 2011; Wilhelm et al., 2014; Schostag et al., 2015). However, these studies sequenced small subunit (SSU) rRNA amplicons derived from reverse-transcribed RNA, and this method is also dependent on “universal” primers for PCR amplification same as DNA based analyses. The limitations inherent to PCR primer-based methods prevent these amplification strategies from being used to correctly characterize microbial communities, especially regarding concurrent quantitative analysis related to the three-domain system of life (Urich et al., 2008). In addition, a large fraction of microbial taxa, known as the “shadow biosphere,” would escape detection due to the use of the so-called “universal” primers (Mao et al., 2012; Youssef et al., 2015).

“Double RNA” metatranscriptomics (which involves studying microbial community transcripts, including rRNA and mRNA, in a particular environment) may provide a more immediate picture of microbial responses to changing conditions by providing both functional and taxonomic information on the microbes (Urich et al., 2008). So far, this metatranscriptomic method has been applied in several microbial community studies (Yu and Zhang, 2012; Hultman et al., 2015; Kim and Liesack, 2015; Zifcakova et al., 2016). The metatranscriptome is typically derived from the reverse-transcription of RNA using random primers and hence it avoids the drawbacks of PCR amplification.

However, comparisons of microbial communities based on operational taxonomic unit (OTU) clustering have never previously been performed because of the regional variations in the random primer-derived SSU rRNA sequences. Although diversity indices could be calculated and compared using the V3 region of 16S rRNA sequences when enriched SSU rRNA is analyzed, only a third of the resulting 16S rRNA sequences were found to be suitable for such analyses (Li et al., 2014). In addition, using this method, high quantities of purified RNA were usually required for library preparation (Li et al., 2014; McDonald et al., 2016).

Recently, we developed a modified metatranscriptomic method that requires an immediate adaptor ligation step at the 5′ end of the RNA before it undergoes reverse-transcription. Using this method, we could obtain more 16S rRNA sequences of same regions (V1–V2) without the interference of DNA in order to analyze OTU-based microbial communities and diversity (Yan et al., 2017). In addition, the construction of a total nucleic acid library based on diverse environmental samples, especially low-biomass samples with low RNA yields, was shown to be feasible (Yan et al., 2017).

The aims of this study were (i) to obtain a detailed view of the depth-wise dynamics of potentially active microbial communities and their diversities in mudflat sediments from Chongming Island using our modified metatranscriptomic method, so as to improve our understanding of the biogeochemical processes within this complex ecosystem, (ii) to discover certain potentially active taxa that generally escape detection by PCR amplification of 16S rRNA genes, and (iii) to evaluate the taxon-specific biases that are generated by the PCR-based methods through paired comparisons using datasets of metatranscriptome and 16S rRNA gene amplicons based on both DNA and cDNA.

## Materials and Methods

### Sample Collection and Measurements of Physicochemical Parameters

Core sediment samples were collected from the intertidal mudflat of northern Dongtan on Chongming Island (121°57′E, 31°33′N). Sampling was carried out on October 7th, 2015. Three sampling locations (S1–S3) that were approximately 20 m apart were selected as biological replicates, and three replication points were used for each location. The sediment samples were collected using short core samplers (internal diameter, 5.0 cm; total length, 0.5 m), and the samples were then segmented into four depth-wise samples (0–1, 1–5, 5–15, and 15–40 cm; giving 3 × 3 × 4 = 36 samples).

Sediment temperature, electrical conductivity (EC), and oxidation-reduction potential (ORP) were measured immediately, while the samples were in the core sampler. Temperature and EC were measured directly by inserting a Waterproof Ectestr11+ sensor (Thermo Fisher Scientific, Waltham, MA, United States) into the samples to read both parameters. ORP was measured with the Bante211 portable pH/ORP meter (Bante, Shanghai, China) using the same method. Immediately after taking the measurements, half of each set of three replicate samples was homogenized with the corresponding location and depth (generating 3 × 4 = 12 homogenized samples). Subsequently, 5 g of each homogenized samples was stored in 10 mL LifeGuard Soil Preservation Solution (Mo Bio, Carlsbad, CA, United States) for RNA extraction and the remaining amounts were used for DNA extraction. All the samples were stored in 50 mL DNase/RNase-free sterile centrifuge tubes or sterile polypropylene bags and transported to the laboratory using an icebox. Samples for the chemical analysis were stored at 4°C. Samples for the RNA-based molecular analysis were stored at -80°C, whereas samples for the DNA-based analysis were stored at -20°C.

Regarding the chemical analysis, all the measurements were carried out according to Zeleke et al. (2013) and Hultman et al. (2015) with slight modifications. Briefly, for each sample, 20 g sediment was completely dried at 50°C overnight, and rock particles and root materials were removed from the gently ground sediments. Dried sediments were ground into powder and sifted through 0.25-mm mesh sieves. Parts of the powder were used for the determination of both total C and N content using an NC analyzer (FlashEA1112 Series, Thermo Fisher Scientific, Bremen, Germany). Suspensions were prepared from 10 g sediment powder from each sample by adding 40 mL deionized water. Supernatants were filtered using 0.22-μm filters (Sangon, Shanghai, China) after shaking and settlement of the suspensions. For each sample, 5 mL of the clear supernatant was used to determine the sulfate ion concentrations by ion chromatography (ICS-1000, Dionex, Sunnyvale, CA, United States), and the sulfate content for each gram of dry weight soil (DWS) was then calculated. The pH of the remaining supernatants was measured with a Bante211 portable pH/ORP meter (Bante, Shanghai, China).

### Nucleic Acid Extraction

For each sample, the total RNA was isolated from 2 g sediment using a PowerSoil RNA Isolation Kit (Mo Bio, Carlsbad, CA, United States), and it was stored at -80°C prior to the next step. Genomic DNA from the sediment samples was extracted using a PowerSoil DNA Isolation Kit (Mo Bio, Carlsbad, CA, United States) according to the manufacturer’s instructions. Extracted DNA was stored at -20°C until amplification. RNA and DNA quality and integrity was assessed by gel electrophoresis.

The total RNA was quantified using a Qubit RNA Assay Kit with a Qubit 2.0 fluorometer (Life Technologies, Carlsbad, CA, United States) and then used for preparation of the metatranscriptome libraries.

### cDNA Synthesis

For the cDNA amplicon analyses, the total RNA from the S2 location samples was first digested with DNase I (TaKaRa, Dalian, China) for 1 h and then purified using a MinElute Cleanup Kit (Qiagen, Hilden, Germany). The absence of residual genomic DNA was checked by 30-cycle PCR amplification of the purified RNA using the prokaryotic universal primer set Pro341F and Pro805R (Bourtzis et al., 2014). Amplification was performed with an initial denaturation at 94°C for 5 min followed by 30 cycles of 94°C for 30 s, 55°C for 30 s, and 72°C for 30 s, and a final extension at 72°C for 7 min. The first strand of cDNA was synthesized using random hexamers and a Goscript cDNA Synthesis System (Promega, Madison, WI, United States) according to the manufacturer’s protocol.

### Preparation of Metatranscriptome Libraries

A preliminary experiment had previously been designed and conducted to confirm that residual genomic DNA has no effect on library construction and data analysis even if the quantity of inputted DNA was several times higher than that of the RNA (Yan et al., 2017). Therefore, each metatranscriptome library was constructed directly using 20 ng total RNA without a residual DNA removal step. All the libraries were prepared using a modified version of the RNA-seq Library Preparation Kit protocol (Gnomegen, San Diego, CA, United States). Total RNA was heat-denatured at 65°C for 5 min instead of fragmentation at 95°C as the protocol suggests. Next, an RNA-seq 5′ adaptor was directly ligated to the 5′ end of the heat-denatured full-length RNA at 37°C for 2 h. After the ligation, the products were purified using a Gnome Size Selector (Gnomegen, San Diego, CA, United States) and the first strand of cDNA was synthesized from the products with a tagged random hexamer. The synthesized cDNA was also purified using a Gnome Size Selector according to standard instructions. To enrich the products for sequencing, 15 cycles of PCR amplification were performed on the first cDNA strands using Illumina-compatible primer sets. These primers were designed according to the adaptor and tag sequences and were complementary to the standard Illumina forward and reverse primers. The reverse primer also contained an 8-nucleotide (nt) indexing sequence to allow for multiplexing. The 400–600-base pair (bp) PCR products were size-selected using a Gnome Size Selector and sequenced on an Illumina MiSeq platform using the 2 × 300 paired-end protocol.

### Amplicon Library Preparation

A total of 12 DNA samples (for all three locations) and four cDNA samples (for the S2 location only) were amplified in 50-μL reaction systems using the primer sets of barcoded Pro341F (5′-XXXXXXXXCCTACGGGNBGCASCAG-3′, XXXXXXXX denotes the barcode sequence) and Pro805R (5′-GACTACNVGGGTATCTAATCC-3′) (Bourtzis et al., 2014) under the aforementioned conditions. For each sample, three replicates of both 16S rDNA and 16S cDNA amplicons were pooled and purified using an AxyPrep DNA Gel Extraction Kit (Axygen, Tewksbury, MA, United States). The amplicon DNA concentrations were measured using a Qubit dsDNA HS Assay Kit with a Qubit 2.0 fluorometer (Life Technologies, Carlsbad, CA, United States). Based on the quantification results, the cleaned amplicons were mixed at equimolar ratios for library construction. Using a KAPA LTP Library Kit (KAPA Biosystems, Boston, MA, United States), end-repairing, amplicon A-tailing, and adaptor ligating were carried out according to standard protocols. After ligation of the sequencing adaptors, each composite library was cleaned again with an AxyPrep DNA Gel Extraction Kit (Axygen, Tewksbury, MA, United States). Next-generation sequencing was performed on an Illumina MiSeq platform using the 2 × 300 protocol.

### Bioinformatic Analysis

Sequences were processed mainly using software QIIME v1.8.0 (Caporaso et al., 2010), mothur v1.33.3 (Schloss et al., 2009), and Usearch v8.1 (Edgar, 2010). Pair-end reads were pre-processed with Sickle software v1.33 (Joshi and Fass, 2011) to trim and filter reads with Phred quality score <20. Joining of paired-end reads was conducted with the “join_paired_ends.py” command in QIIME v1.8.0. Sequences with any ambiguous nucleotide and homopolymer length >8 bases were removed from the assembled sequences in mothur v1.33.3.

For each metatranscriptome (MT) dataset, sequences >250 bp were submitted to MIPE software^1^ for identification of archaeal, bacterial, and eukaryotic SSU rRNA (denoted as “MT-SSU”), with a bootstrap cut-off of 80% against the SILVA SSU seed v119 database^2^. Identified prokaryotic SSU rRNA reads (denoted as “MT-16S”) were aligned with the online alignment tool (Aligner^3^) and trimmed in order to leave the region 8F-V1-V2 (*Escherichia coli* positions 8–242) using the “pcr.seqs” command in mothur. The pre-processed sequences were then merged according to datasets and subjected to OTU-related analysis as amplicon datasets, while the taxonomy assignment for each representative OTU sequence was carried out using a minimum bootstrap confidence of 50% against the SILVA SSU nr_v119 database^2^.

For the 16S rRNA gene amplicon datasets, reads were assigned to libraries according to the 8-nt barcodes based on a quality value >20. Sequences >300 bp were processed in Usearch v8.1 for OTU clustering based on 97% identity. Singletons and chimeric sequences were removed from the analysis. Taxonomic information was assigned to each representative OTU sequence using a minimum bootstrap confidence of 80% against the SILVA SSU nr_v119 database. Unassigned sequences or sequences belonging to chloroplasts and mitochondria were discarded. For each sample, the proportion of each taxon and alpha diversity indices were calculated in QIIME. For each dataset, 5000 randomly selected sequences were used for the calculation of alpha diversity indices, which involved observed OTUs, Chao1, Shannon, and phylogenetic distance (PD).

BIOM files of the MT-16S and amplicon datasets, derived from the OTU clustering analysis and taxonomy assignment, were merged for hierarchical average linkage clustering (to construct an unweighted pair group method with arithmetic mean [UPGMA] tree) in QIIME based on Bray–Curtis distance. For the clustering, 5000 sequences were randomly selected for each sample.

### Statistical Analysis

Pearson’s correlation coefficients between the relative abundances of each taxon and environmental parameters were calculated using the Hmisc package in R (R Core Team, 2009). Taxa with *P*-values < 0.05 were considered to be significantly correlated with the environmental parameters. Non-metric multi-dimensional scaling (NMDS) ordination (based on Bray–Curtis distance) and a redundancy analysis with 999 Monto Carlo permutation tests were also performed in R by employing the vegan package (R Core Team, 2009). The relative abundances of each taxon were compared across datasets of the same sample using STAMP software (Parks and Beiko, 2010), and a two-tailed Fisher’s exact test was performed to determine the significance of differences in the observed relative abundances. A Bonferroni correction was used, and all the between-dataset differences with corrected *P*-values < 0.05 were considered to represent significant differences in the relative abundances of specific taxa. A Wilcoxon signed rank test was performed in R (R Core Team, 2009) to determine the significance of differences in observed relative abundances across datasets for a specific taxon at the location level.

### Nucleotide Sequence Accession Numbers

High-throughput raw sequence data have been deposited in the United States National Center for Biotechnology Information (NCBI) GenBank Short Read Archive (SRA) under the accession numbers SRR5988215–SRR5988242.

## Results

### Physicochemical Parameters of Mudflat Sediments

Physicochemical parameters at different depths in three locations were measured (**Table 1**). The water content (21.7–31.2%, w/w), temperature (25–28°C), and pH (6.80–7.56) were relatively stable across all of the depths. There was a depth-wise reduction in total N and C content. The sulfate concentrations were relatively high at all depths (620.1–1327.4 mg kg^-1^ DWS) and the sulfate concentration seemed to display a decreasing trend with increasing depth, although it was much higher in the lowest sediment sample of S2 (designated “S2-4”). The EC of the upper sediments tended to be lower than that of the deeper sediments. The situation was almost the same for the reductive potentials except for the 5–15 cm sediment sample of S1 (designated “S1-3”).

**Table 1:** Physicochemical soil parameters for the different depths of mudflat sediments (mean ± SD, *n* = 3).

Samples	Temp (°C)	pH	Moisture (%)	EC (mS)	ORP (mV)	TN (%)	TC (%)	Sulfate (mg kg^-1^ DWS)
S1-1^a^	28.4 ± 0.4	6.90 ± 0.15	25.0 ± 0.4	5.13 ± 0.03	–132.5 ± 17.7	0.072 ± 0.006	1.414 ± 0.020	929.3 ± 87.9
S1-2	27.4 ± 1.4	6.80 ± 0.09	23.4 ± 0.3	4.48 ± 0.63	–160.3 ± 5.7	0.071 ± 0.002	1.332 ± 0.022	1087.2 ± 171.1
S1-3	26.2 ± 0.2	7.09 ± 0.2	22.4 ± 0.4	4.26 ± 0.42	–62.3 ± 29.7	0.052 ± 0.008	1.340 ± 0.044	620.1 ± 61.3
S1-4	26.2 ± 0.7	6.84 ± 0.23	24.0 ± 1.2	6.55 ± 0.19	–114.7 ± 25.0	0.057 ± 0.008	1.260 ± 0.089	863.1 ± 189.3
S2-1	28.5 ± 0.6	6.94 ± 0.20	28.3 ± 2.3	5.46 ± 0.30	–111.7 ± 8.5	0.087 ± 0.011	1.331 ± 0.096	899.5 ± 176.8
S2-2	25.3 ± 2.1	6.59 ± 0.07	28.7 ± 0.4	4.33 ± 1.05	–159.7 ± 12.7	0.085 ± 0.003	1.342 ± 0.072	1145 ± 130.9
S2-3	26.1 ± 0.7	7.24 ± 0.14	28.2 ± 1.1	5.07 ± 0.09	–153.7 ± 5.5	0.071 ± 0.006	1.482 ± 0.037	772.0 ± 86.0
S2-4	28.3 ± 0.9	6.71 ± 0.23	31.2 ± 1.3	6.23 ± 0.88	–169.7 ± 30.0	0.057 ± 0.049	1.133 ± 0.984	1327.4 ± 240.4
S3-1	26.3 ± 0.3	7.34 ± 0.09	24.6 ± 0.3	4.21 ± 0.13	–99.5 ± 10.6	0.079 ± 0.008	1.424 ± 0.046	867.2 ± 15.5
S3-2	27.8 ± 0.7	7.52 ± 0.15	22.5 ± 0.3	4.52 ± 1.07	–138.7 ± 11.0	0.077 ± 0.007	1.364 ± 0.096	633.6 ± 16.7
S3-3	27.2 ± 0.1	7.38 ± 0.11	23.4 ± 0.3	5.13 ± 0.07	–125.7 ± 28.6	0.068 ± 0.003	1.302 ± 0.048	742.4 ± 97.9
S3-4	28.6 ± 0.7	7.56 ± 0.22	21.7 ± 0.1	4.34 ± 0.27	–99.3 ± 17.6	0.063 ± 0.006	1.352 ± 0.079	740.2 ± 145.5

*Temp, temperature; Moisture, water content; EC, electrical conductivity; ORP, oxidation-reduction potential; TN, total nitrogen content; TC, total carbon content; Sulfate, sulfate concentration; DWS, dry weight soil. ^*a*^The number after ‘S’ denotes the sampling location (S1, S2, or S3) and the final number (1, 2, 3, or 4) represents the sampling depth (0–1 cm, 1–5 cm, 5–15 cm, or 15–40 cm, respectively).*

### Microbial Diversity and Richness

Microbial community compositions were concurrently characterized based on both the MT-SSU datasets and 16S rDNA amplicons. As revealed by MT-SSU sample datasets, a range of approximately 11586–77649 (mean ± SD: 52313 ± 19699) sequences were identified as prokaryotic SSU rRNA (MT-16S), and more than 60% of these sequences in each sample dataset could be clustered with an identity of 97% into 1964–5691 (4624 ± 1058) OTUs (**Supplementary Table S1**). At the same time, approximately 196–5689 SSU rRNA sequences in each dataset were identified as eukaryotic, which occupied 0.4–20.3% of the MT-SSU datasets. As for the amplicon datasets, the number of prokaryotic 16S rRNA sequences ranged from 5586 to 39737 (19159 ± 8708), belonging to 932–1591 OTUs (1288 ± 174) (**Supplementary Table S2**).

**FIGURE 1:**
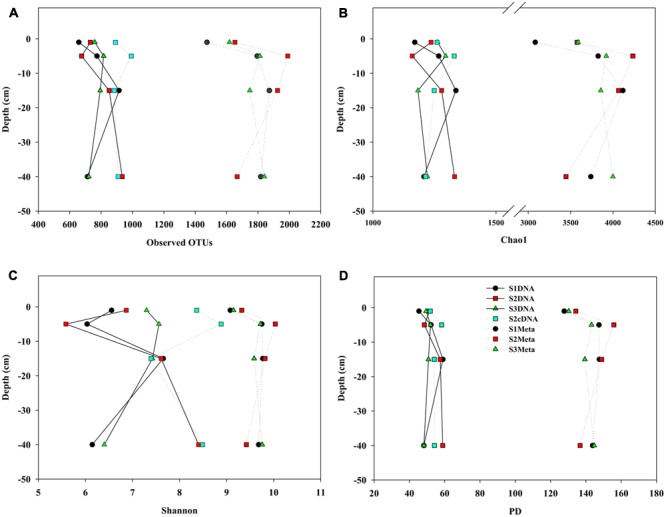
Comparisons of alpha indices for mudflat sediments at different depths derived from amplicon and MT-16S datasets. **(A)** Observed OTU numbers; **(B)** Chao 1; **(C)** Shannon indices; **(D)** PD indices. For each dataset, 5000 sequences were randomly selected for each diversity calculation. S1, S2, and S3 represent the sampling locations; DNA and cDNA indicate 16S rDNA and 16S cDNA amplicon datasets, and Meta indicates MT-16S datasets.

Using 5000 randomly selected 16S rRNA sequences from each sample dataset, alpha diversity indices for sediment samples taken from different depths were calculated and compared (**Figure 1**). From the MT-16S analyses of observed OTUs, Chao1, Shannon, and PD index, the lowest prokaryotic diversity was observed in the surface sediment samples, and high diversity was observed in the second-layer sediment samples (1–5 cm). Although similar trends were also observed for the 16S rDNA amplicons, the indices varied occasionally and were always half or one third the values of those in the MT-16S analyses. 16S cDNA amplicons from location S2 yielded alpha diversity indices much closer to the 16S rDNA amplicons that were amplified with the same primer set.

### Microbial Community Compositions

**FIGURE 2:**
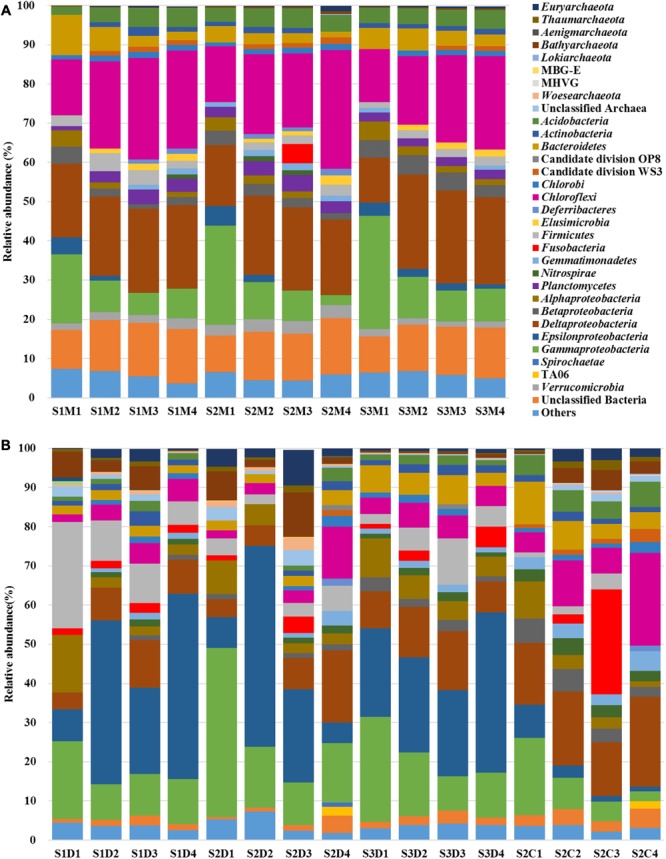
Prokaryotic community structures identified by the datasets of MT-16S **(A)** and amplicons **(B)**. Compositions are illustrated at the level of the phylum or class (for *Proteobacteria*). Archaeal taxa with relative abundance <0.1%, bacterial taxa with relative abundance <1%, and unclassified *Proteobacterial* 16S rRNA gene sequences are classified into “Others.” “Unclassified archaea or bacteria” include 16S rRNA gene sequences under bootstrap cut-off values (80% for amplicons and 50% for MT-16S). *Aenigmarchaeota, Bathyarchaeota, Lokiarchaeota*, MBG-E, MHVG were identified as Deep Sea Euryarchaeotic Group, Miscellaneous Crenarchaeotic Group, Marine Benthic Group B, Marine Benthic Group E and Marine Hydrothermal Vent Group, respectively, in SILVA SSU v119 database. S1, S2, and S3 represent the sampling locations; D, C, and M indicate the 16S datasets of rDNA amplicons, cDNA amplicons, and the metatranscriptome, respectively; 1, 2, 3, and 4 denote the sampling depth (0–1 cm, 1–5 cm, 5–15 cm, and 15–40 cm, respectively).

Both MT-16S and 16S rDNA amplicons revealed the most important fraction of the prokaryotic communities was the bacterial fraction. It accounted for 97.64–99.77% in the MT-16S datasets in contrast to 70.07–98.38% in the 16S rDNA amplicons (**Figure 2**). The archaeal phyla *Euryarchaeota, Thaumarchaeota*, and *Bathyarchaeota* [identified as Miscellaneous Crenarchaeotic Group (MCG) in SILVA SSU v119 database] were also detected in both types of dataset, although archaea occupied a very small proportion of the MT-16S datasets (0.23–2.36%) (**Figure 2A**).

**FIGURE 3:**
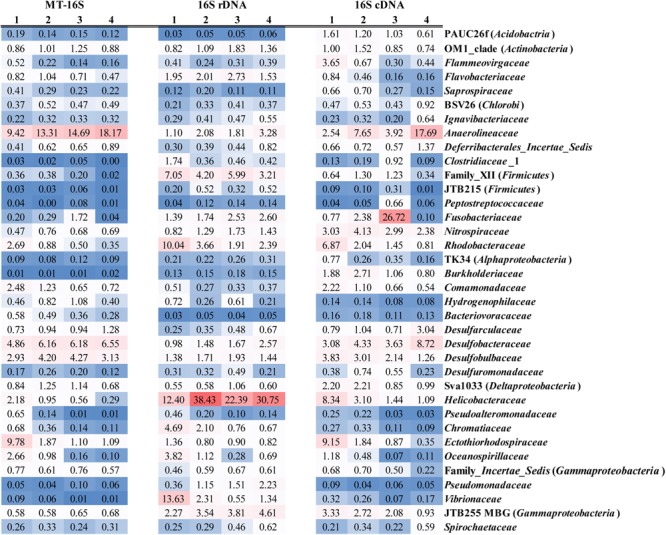
Average relative abundances of bacterial families across all the depths identified by the datasets of MT-16S and both 16S rDNA and cDNA amplicons. Families with average relative abundance <0.5% in all of the datasets are not displayed. Explanations for 1, 2, 3, and 4 are given in **Figure 2**.

Regarding the MT-16S analysis, the dominant potentially active bacterial taxa were assigned to the phylum *Chloroflexi* and the classes *Deltaproteobacteria* and *Gammaproteobacteria* (**Figure 2A**). The family *Anaerolineaceae* dominated the phylum *Chloroflexi*, and its relative abundance increased from 9.42% ± 0.95% in the surface sediments to 18.17% ± 2.81% in the bottom sediments (**Figure 3**). Bacteria in the families *Desulfobacteraceae* (major genus Sva0081 sediment group) and *Desulfobulbaceae* (major genus *Desulfobulbus*), which both belong to the dominant order *Desulfobacterales* and class *Deltaproteobacteria*, were prevalent. The families *Ectothiorhodospiraceae* and *Oceanospirillaceae* in the class *Gammaproteobacteria* were also prevalent. According to our results, *Desulfobacteraceae* (4.86% ± 2.42% to 6.55% ± 0.80%) and *Desulfobulbaceae* (2.93% ± 0.76% to 3.13% ± 1.78%) each displayed a steadily increasing trend as the depths increased, in contrast to the depth-wise decrease in the families *Ectothiorhodospiraceae* (9.78% ± 4.38% to 1.09% ± 0.56%) and *Oceanospirillaceae* (2.66% ± 1.08% to 0.10% ± 0.04%) (**Figure 3**). Two other relatively abundant taxa in the MT-16S datasets were the phyla *Bacteroidetes* and *Acidobacteria* (**Figure 2A**). The abundance of *Bacteroidetes* showed an obvious depth-related reduction (6.61% ± 3.27% to 2.21% ± 0.82%). However, the abundance of *Acidobacteria* displayed a weak depth-related increase, with the relative abundance increasing from 3.25% ± 0.92% in the surface sediments to 4.66% ± 0.32% in the bottom sediments (**Figure 2A**). The potentially active phyla *Elusimicrobia* (0.67% ± 0.08% to 1.98% ± 0.36%), *Lentisphaerae* (0.15% ± 0.11% to 0.25% ± 0.13), *Planctomycetes* (2.02% ± 0.75% to 2.95% ± 0.49%), and *Verrucomicrobia* (2.14% ± 0.63% to 2.57% ± 0.86%) were exclusive to the MT-16S datasets, and all displayed a depth-wise increasing trend, but they constituted a very small fraction of the total datasets (**Supplementary Data Sheets S1, S2**). Although not as abundant as bacteria, the dominant potentially active archaeal groups belonged to the order *Thermoplasmatales* (0.18% ± 0.12% to 0.75% ± 0.59%) and the phylum *Bathyarchaeota* (0.06% ± 0.04% to 0.25% ± 0.19%), and there was a depth-wise increase in their relative abundances (**Supplementary Data Sheet S1**). Regarding the identified eukaryotic SSU rRNA sequences, the diatom-related subphylum *Bacillariophytina* dominated and was highly enriched in all the sediments, with an abundance of 47.46% ± 23.97% in the surface sediments and 57.33% ± 22.75% in the deeper sediments, with no obvious depth-wise trends.

Regarding the 16S rDNA amplicon results, *Gammaproteobacteria* was relatively abundant, with the same distributions as revealed by the metatranscriptomic method (MT-16S) (**Figure 2B**). However, the order *Vibrionales* (13.63% ± 10.89% to 1.34% ± 0.94%) within the class *Gammaproteobacteria* was another prevalent order in addition to the two aforementioned orders (**Supplementary Data Sheet S2**). The abundances of the family *Anaerolineaceae* (1.10% ± 0.65% to 3.28% ± 0.54%) and the order *Desulfobacterales* (2.39% ± 1.20% to 4.06% ± 2.18%) were not as high as they were in the MT-16S datasets, although similar depth-related trends were revealed (**Figure 3**). In addition to *Gammaproteobacteria*, the 16S rDNA amplicon results revealed that the phylum *Firmicutes* and the classes *Alpha-* and *Epsilon-proteobacteria* were also major bacterial taxa for the three locations. The abundances of *Firmicutes* and *Alphaproteobacteria* decreased with depth, while the abundance of *Epsilonproteobacteria* increased (**Figure 2B**). As for archaea, all of the dominant groups that were determined by MT-16S were also abundant according to the 16S rDNA amplicon results, and they occupied many more fractions in the amplicon datasets. However, the order *Methanosarcinales* was relatively abundant in the 16S rDNA amplicons but was rarely detected in the MT-16S analyses (**Supplementary Data Sheets S1, S2**). In addition, the *Woesearchaeota* [identified as deep sea hydrothermal vent group 6 (DHVEG-6) in SILVA v119 database] was another prevalent taxon that was not detected by the metatranscriptomic method, and it exhibited a depth-wise increasing trend (**Supplementary Data Sheet S2**).

A third type of amplification profiling dataset was the 16S cDNA amplicon datasets, which were derived from 16S rRNA amplification of randomly reverse-transcribed RNA and were only employed for four samples from location S2. The bacterial community structures established based on the 16S cDNA amplicons were more similar to those of the MT-16S analyses rather than the 16S rDNA amplicons (**Figures 2, 3**). In contrast, the archaeal communities (notably the most prevalent archaeal taxa) were more consistent with the 16S rDNA amplicon results (**Supplementary Data Sheet S2**).

### Comparison of Prokaryotic Communities According to the Different Types of Datasets

**FIGURE 4:**
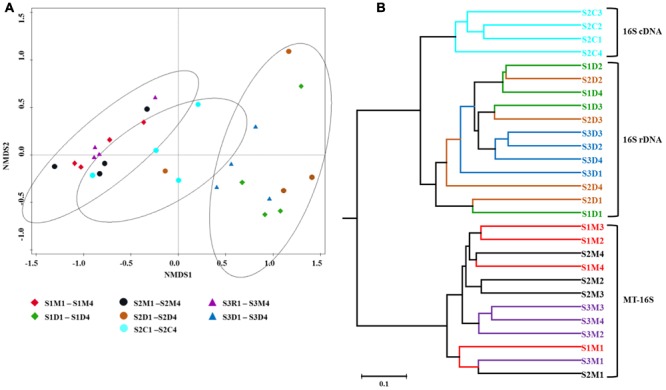
Relationships between individual datasets illustrated by non-metric multi-dimensional scaling (NMDS) ordination **(A)** and hierarchical average linkage clustering (UPGMA tree) **(B)**. Both analyses were performed on the basis of Bray–Curtis dissimilarities between relative taxon abundances. Symbols of the same color indicate datasets of sediment samples from the same location. Explanations for abbreviations of datasets are given in **Figure 2**.

To visualize community differences predicted by the different datasets, Bray–Curtis dissimilarities were calculated utilizing the relative prokaryotic abundances, and an NMDS ordination was carried out and a UPGMA tree was constructed (**Figure 4**). Both analyses illustrated that the communities determined by the MT-16S analyses and both the 16S rDNA and 16S cDNA amplicon analyses were distinctly clustered, and the prokaryotic communities of the top sediment layers were different from those of the deeper layers.

To evaluate the bias generated by the PCR method, all three types of datasets for the S2 location were utilized to compare community differences, notably for the potentially active microbes underestimated using the 16S rDNA amplicon datasets. According to paired comparisons, six main phyla (*Acidobacteria, Chloroflexi, Elusimicrobia, Lentisphaerae, Planctomycetes*, and *Verrucomicrobia*) and one order (*Desulfobacterales*) were determined to be underestimated using the 16S rDNA amplicon datasets across all the depths (**Supplementary Data Sheet S3**). Further paired comparisons between the MT-16S datasets and 16S cDNA amplicon datasets were also performed for these microbial taxa in addition to the comparisons of the 16S cDNA and 16S rDNA amplicon datasets. Three types of abundance underestimation were summarized on the basis of these comparisons (**Table 2** and **Supplementary Table S3**). (i) Underestimation of the potential activity, with higher relative abundances in MT-16S and 16S cDNA amplicons than in 16S rDNA amplicons, e.g., regarding the phylum *Acidobacteria* and orders *Desulfobacterales* and *Myxococcales* (**Table 2**). (ii) Underestimation solely by PCR amplification, with higher relative abundances in MT-16S than in 16S cDNA and 16S rDNA amplicons, e.g., regarding the phyla *Elusimicrobia, Lentisphaerae, Planctomycetes*, and *Verrucomicrobia* (**Table 2**). (iii) A combination of the above two effects, with the relative abundances in MT-16S higher than in 16S cDNA amplicons and the relative abundances in 16S cDNA amplicons also higher than in 16S rDNA amplicons, e.g., regarding the relatively abundant phyla *Chloroflexi* (and notably the prevalent family *Anaerolineaceae*) and *Acidobacteria* (Subgroup 22), and the class *Deltaproteobacteria* (including the family *Bacteriovoracaceae*) (**Table 2**).

**Table 2:** Three types of abundance underestimation based on comparisons among datasets across the four-depth sediments in location S2 for bacterial taxa with higher abundances in MT-16S than 16S rDNA amplicons.

	cDNA (%)^a^	rDNA (%)^b^	MT-16S (%)^c^
	mean ± SD	mean ± SD	mean ± SD
**Type I**			
Phylum			
*Acidobacteria*	5.36 ± 0.78AB	1.61 ± 1.06C	4.45 ± 0.56A
Order			
*Desulfobacterales*	7.54 ± 1.53AB	3.24 ± 1.95C	9.33 ± 0.81A
*Myxococcales*	1.07 ± 0.10AB	0.28 ± 0.14C	1.17 ± 0.12A
**Type II**			
Phylum			
*Elusimicrobia*	0.00 ± 0.00B	0.00 ± 0.00BC	1.28 ± 0.67A
*Lentisphaerae*	0.00 ± 0.00B	0.00 ± 0.00BC	0.30 ± 0.08A
*Planctomycetes*	0.00 ± 0.00B	0.00 ± 0.00BC	3.37 ± 0.59A
*Verrucomicrobia*	0.02 ± 0.02B	0.01 ± 0.00BC	3.15 ± 0.18A
**Type III**			
Phylum			
*Chloroflexi*	11.77 ± 7.29B	5.33 ± 4.62C	20.94 ± 5.89A
Class			
Subgroup 22 (Acidobacteria)	0.82 ± 0.14B	0.21 ± 0.09C	1.63 ± 0.33A
Family			
*Anaerolineaceae*	7.95 ± 5.93B	1.78 ± 1.25C	13.87 ± 4.57A
*Bacteriovoracaceae*	0.15 ± 0.03B	0.03 ± 0.02C	0.39 ± 0.11A

*Different letters (A, B, C) following the abundances indicate a significant difference (*P* < 0.05) based on Wilcoxon signed rank test. ^*a*^Average proportions in 16S cDNA amplicon datasets for the four-depth sediment samples. ^*b*^Average proportions in 16S rDNA amplicon datasets for the four-depth sediment samples. ^*c*^Average proportions in MT-16S datasets for the four-depth sediment samples. **Supplementary Table S3** provides further details.*

### Correlation between Environmental Parameters and Prokaryote Distribution

**FIGURE 5:**
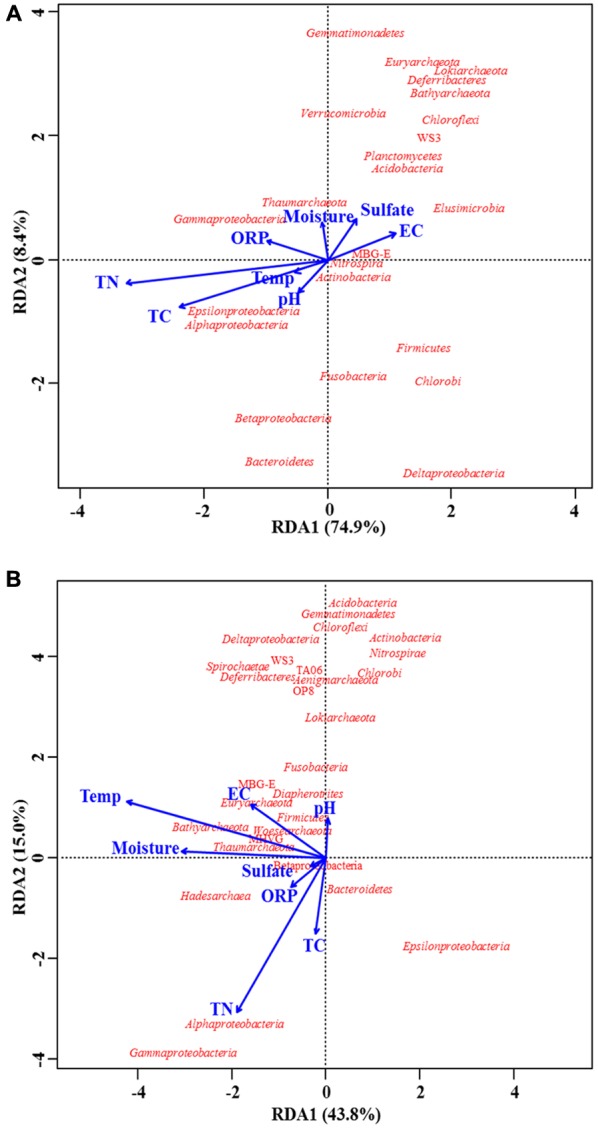
Biplots of redundancy analysis for prokaryotic taxa and environmental parameters. Taxa revealed by both MT-16S **(A)** and 16S rDNA amplicons **(B)** were used for analysis. Correlations are illustrated at the level of the phylum or class (for *Proteobacteria*). Environmental parameters are indicated by blue arrows. Explanations for abbreviations of environmental parameters are given in **Table 1**.

Based on the MT-16S datasets, the redundancy analysis (**Figure 5A**) revealed that the most significant factors associated with microbial distribution were total C and N content, which were also demonstrated by significant Pearson’s correlation coefficients (**Table 3**). For example, the depth-wise reduction in the phylum *Chloroflexi*, dominated by the family *Anaerolineaceae*, was significantly correlated with the depth-wise reduction in both total N and C content (*r* = -0.83, *P* < 0.05 and *r* = -0.69, *P* < 0.05, respectively), while only total N content was significantly correlated with the depth-wise reduction of *Gammaproteobacteria* (*r* = 0.73 and *P* < 0.05). Regarding the minor potentially active phyla, the total N and C content were only significantly correlated with the vertical distribution of *Elusimicrobia* (*r* = -0.79, *P* < 0.05 and *r* = -0.62, *P* < 0.05, respectively). Regarding the two major archaeal groups, only total C content was significantly (negatively) correlated with their vertical distributions. Regarding the potentially active *Deltaproteobacteria, Acidobacteria*, and *Bacteroidetes*, none were significantly affected by any of the environmental factors.

**Table 3:** Significant Pearson’s correlation coefficients between microbial taxa and environmental parameters (*P* < 0.05).

Phylum or class	Temp^a^	pH	Moisture	EC	ORP	TN	TC	Sulfate
**MT-16S**								
*Euryarchaeota*							–0.74	
*Thaumarchaeota*			0.61					
*Bathyarchaeota*							–0.70	
*Lokiarchaeota*							–0.79	
*Actinobacteria*	–0.64							
*Alphaproteobacteria*						0.75	0.60	
*Betaproteobacteria*						0.68		
Candidate_division_WS3						–0.71		
*Chloroflexi*						–0.83	–0.69	
*Elusimicrobia*						–0.79	–0.62	
*Epsilonproteobacteria*						0.79		
*Firmicutes*						–0.60		
*Gammaproteobacteria*						0.73		
*Nitrospirae*	–0.65							
*Planctomycetes*	–0.61							
*Verrucomicrobia*		–0.69	0.84					
**16S rDNA amplicons**								
*Aenigmarchaeota*							–0.74	
*Lokiarchaeota*						–0.61		
*Acidobacteria*						–0.75		
*Actinobacteria*						–0.77		
*Bacteroidetes*		0.62						
*Betaproteobacteria*		0.62						
Candidate_division_OP8							–0.68	
Candidate_division_WS3			0.61				–0.74	0.66
*Chloroflexi*						–0.58	–0.79	
*Deferribacteres*			0.61				–0.74	0.66
*Deltaproteobacteria*							–0.59	
*Gammaproteobacteria*						0.63		
*Gemmatimonadetes*						–0.67	–0.63	
*Nitrospirae*						–0.60		
*Spirochaetae*			0.61				–0.74	0.66
TA06			0.61				–0.74	0.66

*^*a*^Explanations for abbreviations of environmental parameters are given in **Table 1**.*

Although prokaryotic distributions tended to be significantly associated with both total C and N content according to the Pearson’s correlation coefficients (**Table 3**), the redundancy analysis only confirmed the significant associations with total N content for microbes in the 16S rDNA amplicons (**Figure 5B**). In both types of dataset, the environmental impacts on *Chloroflexi* and *Gammaproteobacteria* were the same, but the environmental impacts were very different for other taxa (**Table 3**).

## Discussion

Because of the high content of rRNA and no requirement for specific primers, metatranscriptome sequencing-based SSU rRNA analysis becomes a good option for potentially active microbial community profiling. We modified metatranscriptome method which requires an immediate adaptor ligation step at the 5′ end of the RNA before reverse-transcription. This modified method is capable of constructing metatranscriptome library from total nucleic acid, and the little requirement for RNA quantities enables it can be used for various environmental samples (Yan et al., 2017). The accuracy of this modified method was confirmed with mock communities, although random primers non-randomly hybridize to RNA template and the partial 16S rRNA sequences which only cover the regions of V1–V2 may cause some limitations (Yan et al., 2017).

In this study, we provide insights into the structure and composition of indigenous potentially active microbial communities in the mudflat sediments of northern Dongtan. Being an intertidal wetland ecosystem, northern Dongtan has diverse microbial populations that are responsible for nutrient cycling.

### Microbial Diversities in the Mudflat Sediments

Different depth-related prokaryotic communities were determined and compared based on the RNA- and DNA-based datasets. However, the diversities of potentially active microbes could only be explored based on the SSU rRNA transcripts because rRNA is a marker of potentially active microbes (Blazewicz et al., 2013). In comparison, SSU rRNA genes only reveal the bulk diversities and cannot distinguish between active and inactive microbes (Schostag et al., 2015).

The NMDS and UPGMA analyses revealed community differences between the DNA- and RNA-derived datasets. This finding is supported by results from previous studies (Lanzén et al., 2011; Schostag et al., 2015; Zifcakova et al., 2016). However, in previous studies, alpha diversity indices were rarely compared between datasets because the generated SSU rRNA sequences could not be used for calculations due to random reverse transcription-associated regional variations. The modified metatranscriptome-generated SSU rRNA reads primarily covered the 8F, V1 and V2 regions between *E. coli* positions 8 and 242 and enabled an alpha diversity analysis. Although not comparable to 16S rDNA amplicon datasets from previous studies of intertidal and/or coastal sediments (Wang et al., 2012; Liu et al., 2015), the MT-16S datasets used in this study indicated much higher alpha diversity indices (as much as two or threefold higher) when compared with the 16S rDNA amplicons. This indicates that more potentially active members were involved in the microbial processes in the Dongtan area at the time of sampling than the abundances suggested by the 16S rDNA amplicons. One main reason for underestimation is that a large fraction of the reads (including the unassigned reads detected in the MT-16S analyses) escaped from PCR amplification involving “universal” primers, hence this fraction constituted a significant portion in the “shadow biosphere” (Youssef et al., 2015). This is also evidenced by the fact that the 16S cDNA amplicons generated similar diversities to the 16S rDNA amplicons despite the fact that they were also RNA-derived. Recently, the unassigned reads have been used to identify novel environmental bacterial taxa (Youssef et al., 2012). Therefore, such sequences in our datasets provide many more opportunities to discover novel phylogenetic lineages from northern Dongtan.

### Microbial Activity in the Mudflat Sediments and Their Vertical Distribution Patterns

The numerical dominance of bacteria over archaea in coastal and deep-sea sediments had already been discovered using 16S rDNA amplification (Kimes et al., 2013; Liu et al., 2015). However, the archaeal contribution was overestimated as its abundance in the MT-16S datasets (0.78% ± 0.56%) was generally lower than that indicated by the 16S rRNA gene amplicons (8.58% ± 7.80%). According to the MT-16S datasets, the bacterial phylum *Chloroflexi* and the classes *Deltaproteobacteria* and *Gammaproteobacteria* occupied more than half of all the depth-wise prokaryotic communities, which indicates that they are the most significant contributors to physiological activity within the mudflat sediment layers.

The detection of the prevalent family *Anaerolineaceae* in the phylum *Chloroflexi*, in addition to the relatively abundant phylum *Bacteroidetes* and family *Oceanospirillaceae* in the class *Gammaproteobacteria* demonstrated the depth-wise dynamic activity involving C compound degradation and utilization in Dongtan sediments. Given the higher abundances at the surface of sediments, aerobic *Bacteroidetes* and *Oceanospirillaceae* would be suitable degraders of short-chain organic compounds from tide water or sediment particles (Fernandez-Gomez et al., 2013; Paul et al., 2016). In contrast, *Anaerolineaceae* are well-equipped to perform cellular adhesion and fermentation, though the ecological roles of *Anaerolineaceae* remain understudied (Xia et al., 2016). Therefore, their notable high potential activity in deeper sediments is thought to be mainly responsible for the degradation of long-chain carbohydrates so as to provide substrates for other microbial processes (Liang et al., 2016; Xia et al., 2016). The significant inverse correlation between *Chloroflexi* and depth-related trends in total C content is indicative of organically dependent lifestyles and significant roles in C-related processes. In addition, previous omics studies have also confirmed the potential roles of *Chloroflexi* in locations that are massive reservoirs of C compounds (like mudflats), such as terrestrial aquifer sediments (Hug et al., 2013), thermokarst bog soils (Hultman et al., 2015), and anaerobic digestion sludge (Xia et al., 2016). However, opposite conclusions were once drawn regarding the abundance of *Chloroflexi* in organic-rich intertidal flats based on 16S rDNA amplicon sequencing (Wang et al., 2012; Lv et al., 2016), and these conclusions regarding the scarcity of *Chloroflexi* are supported by our amplicon results. Thus, it is concluded that the PCR-based method for the determination of potential *Chloroflexi* activity was notably affected at the DNA level.

By sequencing 16S rDNA amplicons, the class *Deltaproteobacteria*, which is mainly related to sulfate reduction, has been found to be dominant in sediments from tidal flats in Korea and Hong Kong (Kim et al., 2008; Wang et al., 2012) and north Chinese marginal seas (Liu et al., 2015), and even in marine sediments (Schauer et al., 2009; Wang et al., 2012). As a land–sea interaction area, sediments from Dongtan also have high sulfate concentrations, and are suitable for the growth of sulfate-reducing microbes (Zeleke et al., 2013). However, the potential activity of the microbial communities in Dongtan sediments has never been determined, especially from the angle of RNA. In this study, the high potential microbial activity was confirmed with the help of in-depth MT-16S data. Members of the families *Desulfobacteraceae* and *Desulfobulbaceae* within the order *Desulfobacterales* are well known sulfate-reducing bacteria (Leloup et al., 2005; Gittel et al., 2008), and the depth-related increases in abundance in this study indicate that sulfate reduction frequently occurs in the mudflat sediments, and that this process was highly active in the anaerobic lower sediments. In addition, this activity was also much higher than the total activity contributed by archaea. Therefore, a previous quantification analysis (which was carried out at the DNA level) indicating that methanogenesis was highly important clearly underestimated the potential roles of sulfate-reducing bacteria (Zeleke et al., 2013), let alone the roles of *Anaerolineaceae*, which may also be involved in sulfate reduction due to the discovery of related functional genes (Hultman et al., 2015). On the other hand, bacteria in the family *Ectothiorhodospiraceae* within the class *Gammaproteobacteria*, which are capable of aerobically oxidizing sulfur in inorganic sulfur compounds (Paul et al., 2016), have also been found to function with a relatively high potential activity, especially in surface sediments, and their prevalence has been confirmed in tidal flats (Wang et al., 2012). Taking these results together, the mechanism leading to the insensitivity of sulfate-reducing bacteria to vertical changes in sulfate concentrations remains confusing. However, the co-occurrence of sulfur-oxidizing and sulfate-reducing bacterial activity indicates the existence of a functionally linked intercycle coupling between C and S, as *Desulfobacterales* members are also capable of anaerobic hydrocarbon degradation (Kniemeyer et al., 2007).

The phyla *Elusimicrobia, Lentisphaerae, Planctomycetes*, and *Verrucomicrobia* were exclusive to the MT-16S datasets rather than the 16S rDNA or cDNA amplicons. The phylum *Elusimicrobia* (formerly known as Termite Group I) has been shown to be widespread across environments (Herlemann et al., 2009), while *Lentisphaerae, Planctomycetes*, and *Verrucomicrobia* are clustered in the *Planctomycetes, Verrucomicrobia, Chlamydiae* (PVC) superphylum due to their characteristics and the roles they play in many areas of life (Gupta et al., 2012). The importance of the phyla *Planctomycetes* and *Verrucomicrobia* in global nutrient cycles is now well-known in terms of their functions as anaerobic ammonia oxidizers and acidophilic methane oxidizers, respectively (Strous et al., 1999; Dunfield et al., 2007). However, the low coverage of amplicon universal primers for these two phyla was previously highlighted (Mao et al., 2012), which indicates that the contribution of these phyla in different ecosystems may be underestimated. Although the ecological contributions of microbes in the phyla *Elusimicrobia* and *Lentisphaerae* are not well understood due to the limited numbers of isolates and genome data, the contributions of active microbes from these phyla should be carefully considered in microbial community analyses.

Although occupying small fractions, prevalent archaeal groups also seem to play important roles in C cycling in the deeper Dongtan sediments. The new archaeal phylum *Bathyarchaeota*, formerly known as MCG (Meng et al., 2013), is a core generalist group in the sediment realm, and it is responsible for the anaerobic assimilation of organic C (Fillol et al., 2015). In addition, potentially active members of the order *Thermoplasmatales* have been proposed to have methanogenic lifestyles (Paul et al., 2012; Poulsen et al., 2013). Recently, this archaeal group has been determined to be abundant in hydrothermal sediments using 16S rRNA gene clones (Biddle et al., 2011), but metatranscriptomic analysis only proved its dominance in bovine rumens (Poulsen et al., 2013). Although their potential activity was not numerically comparable to that of other bacteria across all depths, it is possible that *Thermoplasmatales* is the main producer of methane in Dongtan, as a high flux of this greenhouse gas was detected during the same season (Wang et al., 2009).

### Evaluation of PCR Bias for Specific Taxa

As rRNA abundance is determined by both cell abundance and ribosomal number, rRNA abundance has been used as an indicator of the potential physiological activity of the main microbial guilds (Rosselli et al., 2016). 16S cDNA amplicons, derived from randomly reverse-transcribed RNA, were amplified with the same primer set as the 16S rDNA amplicons. Differences revealed by the cDNA and DNA amplicons reflect the differences in the potential activity of the taxa being compared. In contrast, differences revealed by the cDNA amplicons and MT-16S datasets, derived from randomly reverse-transcribed RNA, reflect the bias caused by PCR amplification using a prokaryotic “universal” primer set. Therefore, PCR bias mainly occurred within taxa that are summarized as being associated with type II and III underestimations (**Table 2** and **Supplementary Table S3**).

Bias caused by PCR itself may have resulted from multiple drawbacks of the technique, such as mismatches and low coverage of the “universal” primers (Bru et al., 2008; Mao et al., 2012), GC content of degenerate primers for amplification (Polz and Cavanaugh, 1998; Lanzén et al., 2011), and GC content of target templates (Reysenbach et al., 1992; Wintzingerode et al., 1997). No matter what the causes are, however, the aforementioned physiological activity contributions of all the taxa that were found to be underestimated (notably regarding prevalent *Anaerolineaceae* and *Desulfobacterales*) should be much higher than the contributions indicated by the DNA amplicons, even the cDNA amplicons. It is also believed that the actual potential activity of these microbes reported in previous environmental studies has, to a large extent, also suffered similar underestimations, particularly regarding studies that used PCR-based methods.

## Conclusion

We present the first depth-wise study (using a modified metatranscriptomic method) of potentially active microbial communities in an intertidal mudflat in Dongtan, Chongming Island. The MT-16S datasets revealed that there was two or threefold higher diversity compared with the diversity based on the 16S rRNA gene amplicons. The metatranscriptomic data revealed more microbial groups (possibly involved in C-, N-, and S-related processes) in terms of increased prevalences of the archaea *Bathyarchaeota* and *Thermoplasmatales* and the bacteria *Chloroflexi, Delta-*, and *Gamma-proteobacteria*, indicating the existence of complex biogeochemical interactions in Dongtan. The depth-wise activity distributions of the prevalent microbes appeared to be significantly determined by the N and C content in the sediments. It should be noted that the PCR-based method may underestimate the activity of some taxa, particularly regarding the prevalent taxa *Anaerolineaceae* and *Desulfobacterales*. Virtual microbial processes still await specific functional characterization and geochemical studies. To understand the issues more thoroughly, further locations need to be studied over several seasonal cycles.

## Author Contributions

Y-WY and Z-XQ designed the experiments. Y-WY, Q-YJ, J-GW, and TZ conducted the experiments. Y-WY, TZ, and BZ carried out the microbial analysis. Q-FQ gave suggestions for the experiments and results analysis. Y-WY and Z-XQ prepared the manuscript. All authors were involved in revision of the manuscript and approved its final version.

## Conflict of Interest Statement

The authors declare that the research was conducted in the absence of any commercial or financial relationships that could be construed as a potential conflict of interest.

## Footnotes

1 https://github.com/zoubinok/MIPE

2 https://www.mothur.org/wiki/Silva_reference_files

3 http://pyro.cme.msu.edu

## Supplementary Material

TABLE S1 General statistics for analyzed SSU rRNA sequences and OTUs of MiSeq sequencing metatranscriptome.

TABLE S2 General statistics for analyzed 16S rRNA gene sequences and OTUs of MiSeq sequencing amplicons.

TABLE S3 Three types of abundance underestimation based on comparisons among datasets across the four-depth sediments in location S2 for bacterial taxa with higher abundances in MT-16S than 16S rDNA amplicons.

DATA SHEET S1 All the relative abundances of major archaeal, bacterial and eukaryotic taxa revealed by MT-SSU. Explanations for abbreviations of datasets are given in **Figure 2**.

DATA SHEET S2 All the relative abundances of major archaeal and bacterial taxa revealed by 16S rDNA or cDNA amplicons. Explanations of dataset abbreviations are given in **Figure 2**.

DATA SHEET S3 Detailed abundance comparisons among datasets across the four-depth sediments in location S2 for bacterial taxa with higher abundances in MT-16S than 16S rDNA amplicons. Explanations for abbreviations of samples are given in **Table 1**. Explanations for abbreviations of datasets are given in **Table 2**.
