# Supplementary material for: Microbial Communities and Diversities in Mudflat Sediments Analyzed Using a Modified Metatranscriptomic Method
Source: Front Microbiol. 2018 Jan 31;9:93. doi: 10.3389/fmicb.2018.00093 (PMC5797801; doi:10.3389/fmicb.2018.00093)
Supplement: TABLE S1 — General statistics for analyzed SSU rRNA sequences and OTUs of MiSeq sequencing metatranscriptome. [file Table_1.DOCX]

Table S1 General statistics for analyzed SSU rRNA sequences and OTUs of MiSeq sequencing metatranscriptome.

|  |  |  |  | **MIPE** ^b^ | | | |  | **OTU clustering (97% identity)** | | | |
| --- | --- | --- | --- | --- | --- | --- | --- | --- | --- | --- | --- | --- |
| **Samples** ^a^ | **Assembled** | **After QC** |  | **Total**  **SSU** | **Archaea** | **Bacteria** | **Eukaryota** |  | **Archaea OTUs** | **Bacteria OTUs** | **Archaea reads** | **Bacteria reads** |
| **S1M1** | 38579 | 32954 |  | 14538 | 48 | 11538 | 2952 |  | 12 | 1952 | 19 | 8192 |
| **S1M2** | 81781 | 71910 |  | 39229 | 534 | 37872 | 823 |  | 67 | 3998 | 116 | 23781 |
| **S1M3** | 157307 | 120797 |  | 71137 | 938 | 69892 | 307 |  | 88 | 5586 | 233 | 43445 |
| **S1M4** | 68044 | 59258 |  | 38423 | 449 | 37778 | 196 |  | 54 | 4167 | 129 | 24801 |
| **S2M1** | 143812 | 107640 |  | 41260 | 296 | 36478 | 4486 |  | 47 | 3882 | 121 | 23815 |
| **S2M2** | 131297 | 97535 |  | 47870 | 634 | 45366 | 1870 |  | 82 | 4938 | 191 | 28229 |
| **S2M3** | 132287 | 100707 |  | 54652 | 680 | 52839 | 1133 |  | 82 | 5058 | 212 | 32976 |
| **S2M4** | 124664 | 98532 |  | 58771 | 2019 | 56295 | 457 |  | 125 | 4377 | 827 | 34218 |
| **S3M1** | 224998 | 127862 |  | 55009 | 575 | 48745 | 5689 |  | 66 | 4256 | 176 | 30463 |
| **S3M2** | 209121 | 150583 |  | 80862 | 1017 | 76632 | 3213 |  | 86 | 5605 | 311 | 46878 |
| **S3M3** | 155804 | 122740 |  | 72847 | 1286 | 71220 | 341 |  | 104 | 5235 | 456 | 43527 |
| **S3M4** | 164881 | 127147 |  | 75162 | 1383 | 73239 | 540 |  | 112 | 5514 | 538 | 44413 |
| **mean** ±**s.d.** |  |  |  |  | 52313 ± 19699 | |  |  | 4624 ± 1058 | | 32339 ± 11477 | |

^a^ S1, 2, and 3 represent the sampling locations; M indicates the 16S rRNA gene datasets derived from metatranscriptome sequencing;

1, 2, 3, and 4 denote sampling depths of 0‒1 cm, 1‒5 cm, 5‒15 cm, and 15‒40 cm, respectively.

^b^ Archaeal, bacterial, and eukaryotic SSU rRNA identified using MIPE software with a bootstrap cut-off of 80% against the SILVA SSU seed v119 database.
