# Supplementary material for: Microbial Communities and Diversities in Mudflat Sediments Analyzed Using a Modified Metatranscriptomic Method
Source: Front Microbiol. 2018 Jan 31;9:93. doi: 10.3389/fmicb.2018.00093 (PMC5797801; doi:10.3389/fmicb.2018.00093)
Supplement: TABLE S2 — General statistics for analyzed 16S rRNA gene sequences and OTUs of MiSeq sequencing amplicons. [file Table_2.DOCX]

Table S2 General statistics for analyzed 16S rRNA gene sequences and OTUs of MiSeq sequencing amplicons

|  |  |  |  | **OTU clustering (97% identity)** | | | |
| --- | --- | --- | --- | --- | --- | --- | --- |
| **Samples** ^a^ | **Assembled** | **After QC** |  | **Archaea**  **OTUs** | **Bacteria**  **OTUs** | **Archaea**  **reads** | **Bacteria**  **reads** |
| **S1D1** | 47273 | 47263 |  | 245 | 1082 | 3632 | 25678 |
| **S1D2** | 61957 | 61945 |  | 285 | 1306 | 3093 | 36644 |
| **S1D3** | 54478 | 54466 |  | 299 | 1336 | 4298 | 27682 |
| **S1D4** | 24121 | 24116 |  | 89 | 1013 | 171 | 13512 |
| **S2D1** | 26457 | 26456 |  | 258 | 875 | 2514 | 11147 |
| **S2C1** | 29711 | 29705 |  | 49 | 883 | 96 | 5490 |
| **S2D2** | 30363 | 30356 |  | 240 | 985 | 1248 | 17760 |
| **S2C2** | 30662 | 30654 |  | 156 | 1098 | 1147 | 9612 |
| **S2D3** | 39498 | 39493 |  | 335 | 1138 | 6736 | 15771 |
| **S2C3** | 27796 | 27786 |  | 164 | 1037 | 1657 | 10532 |
| **S2D4** | 24713 | 24704 |  | 141 | 1144 | 663 | 12709 |
| **S2C4** | 36900 | 36890 |  | 148 | 1145 | 1362 | 14633 |
| **S3D1** | 28878 | 28870 |  | 129 | 1139 | 299 | 18213 |
| **S3D2** | 28468 | 28464 |  | 124 | 1180 | 307 | 17606 |
| **S3D3** | 36583 | 36573 |  | 135 | 1222 | 430 | 23752 |
| **S3D4** | 25637 | 25634 |  | 159 | 1068 | 537 | 17607 |
| **mean ± s.d.** |  |  |  | 1288 ± 174 | | 19159 ± 8708 | |

^a^ S1, 2, and 3 represent the sampling locations; D and C indicate the 16S rRNA gene datasets of 16S rDNA and 16S cDNA amplicons respectively; 1, 2, 3, and 4 denote sampling depths of 0‒1 cm, 1‒5 cm, 5‒15 cm, and 15‒40 cm, respectively.
