# Supplementary material for: Microbial Communities and Diversities in Mudflat Sediments Analyzed Using a Modified Metatranscriptomic Method
Source: Front Microbiol. 2018 Jan 31;9:93. doi: 10.3389/fmicb.2018.00093 (PMC5797801; doi:10.3389/fmicb.2018.00093)
Supplement: TABLE S3 — Three types of abundance underestimation based on comparisons among datasets across the four-depth sediments in location S2 for bacterial taxa with higher abundances in MT-16S than 16S rDNA amplicons. [file Table_3.DOCX]

Table S3. Three types of abundance underestimation based on comparisons among datasets across the four-depth sediments in location S2 for bacterial taxa with higher abundances in MT-16S than 16S rDNA amplicons.

|  | | **cDNA (%) ^a^** | **rDNA (%) ^b^** | **MT-16S (%) ^c^** |
| --- | --- | --- | --- | --- |
|  | | **mean ± s.d.** | **mean ± s.d.** | **mean ± s.d.** |
| **Type I** | |  |  |  |
| **Phylum** | |  |  |  |
| *Acidobacteria* | | 5.36 ± 0.78AB | 1.61 ± 1.06C | 4.45 ± 0.56A |
| **Order** | |  |  |  |
| *Desulfobacterales* | | 7.54 ± 1.53AB | 3.24 ± 1.95C | 9.33 ± 0.81A |
| *Myxococcales* | | 1.07 ± 0.10AB | 0.28 ± 0.14C | 1.17 ± 0.12A |
| **Family** | |  |  |  |
| *Desulfobacteraceae* | | 4.94 ± 2.23AB | 1.93 ± 1.72C | 5.76 ± 1.16A |
| MSB-4B10 | | 0.13 ± 0.06AB | 0.01 ± 0.01C | 0.21 ± 0.04A |
| **Genus** | |  |  |  |
| Sva0081 sediment  group | | 2.71 ± 1.39AB | 0.79 ± 0.72C | 2.56 ± 0.61A |
| **Type II** | |  |  |  |
| **Phylum** | |  |  |  |
| *Elusimicrobia* | | 0.00 ± 0.00B | 0.00 ± 0.00BC | 1.28 ± 0.67A |
| *Lentisphaerae* | | 0.00 ± 0.00B | 0.00 ± 0.00BC | 0.30 ± 0.08A |
| *Planctomycetes* | | 0.00 ± 0.00B | 0.00 ± 0.00BC | 3.37 ± 0.59A |
| *Verrucomicrobia* | | 0.02 ± 0.02B | 0.01 ± 0.00BC | 3.15 ± 0.18A |
| **Class** | |  |  |  |
| *Caldilineae* | | 0.00 ± 0.00B | 0.00 ± 0.00 BC | 0.17 ± 0.04A |
| *Elusimicrobia* | | 0.00 ± 0.00B | 0.00 ± 0.00 BC | 1.49 ± 0.64A |
| *Holophagae* | | 0.28 ± 0.13B | 0.44 ± 0.25 BC | 1.29 ± 0.05A |
| OM190 | | 0.00 ± 0.00B | 0.00 ± 0.00 BC | 0.57 ± 0.14A |
| OPB35 soil group | | 0.01 ± 0.01B | 0.00 ± 0.00 BC | 2.71 ± 0.23A |
| *Phycisphaerae* | | 0.00 ± 0.00B | 0.00 ± 0.00 BC | 1.69 ± 0.36A |
| Pla3 lineage | | 0.00 ± 0.00B | 0.00 ± 0.00 BC | 0.16 ± 0.03A |
| *Planctomycetacia* | | 0.00 ± 0.00B | 0.00 ± 0.00 BC | 0.42 ± 0.11A |
| R76-B128 | | 0.00 ± 0.00B | 0.00 ± 0.00 BC | 0.13 ± 0.04A |
| vadinHA49 | | 0.00 ± 0.00B | 0.00 ± 0.00 BC | 0.12 ± 0.04A |
| **Order** | |  |  |  |
| *Caldilineales* | | 0.00 ± 0.00B | 0.00 ± 0.00 BC | 0.17 ± 0.04A |
| CCM11a | | 0.00 ± 0.00B | 0.00 ± 0.00 BC | 0.28 ± 0.07A |
| MSBL9 | | 0.00 ± 0.00B | 0.00 ± 0.00 BC | 0.52 ± 0.13A |
| *Phycisphaerales* | | 0.00 ± 0.00B | 0.00 ± 0.00 BC | 0.61 ± 0.22A |
| *Planctomycetales* | | 0.00 ± 0.00B | 0.00 ± 0.00 BC | 0.41 ± 0.11A |
| Rs-M47 | | 0.00 ± 0.00B | 0.00 ± 0.00 BC | 1.27 ± 0.67A |
| Subgroup 23 | | 0.17 ± 0.10B | 0.21 ± 0.21 BC | 0.82 ± 0.15A |
| **Family** | |  |  |  |
| *Caldilineaceae* | | 0.00 ± 0.00B | 0.00 ± 0.00 BC | 0.17 ± 0.04A |
| *Phycisphaeraceae* | | 0.00 ± 0.00B | 0.00 ± 0.00 BC | 0.52 ± 0.21A |
| *Planctomycetaceae* | | 0.00 ± 0.00B | 0.00 ± 0.00 BC | 0.41 ± 0.11A |
| **Genus** | |  |  |  |
| *Desulfobacula* | | 0.00 ± 0.00B | 0.00 ± 0.00 BC | 0.24 ± 0.08A |
| *Desulfopila* | | 0.02 ± 0.01B | 0.01 ± 0.01 BC | 1.06 ± 0.49A |
| *Ornatilinea* | | 0.00 ± 0.00B | 0.00 ± 0.00 BC | 0.24 ± 0.04A |
| Urania-1B-19 marine sediment group | | 0.00 ± 0.00B | 0.00 ± 0.00 BC | 0.34 ± 0.14A |
| **Type III** |  | |  |  |
| **Phylum** |  | |  |  |
| *Chloroflexi* | 11.77 ± 7.29B | | 5.33 ± 4.62C | 20.94 ± 5.89A |
| **Class** |  | |  |  |
| *Anaerolineae* | 7.95 ± 5.93B | | 1.78 ± 1.25C | 13.87 ± 4.57A |
| *Ardenticatenia* | 0.54 ± 0.19B | | 0.15 ± 0.06C | 3.55 ± 0.46A |
| Subgroup 22 | 0.82 ± 0.14B | | 0.21 ± 0.09C | 1.63 ± 0.33A |
| **Order** |  | |  |  |
| *Anaerolineales* | 7.95 ± 5.93B | | 1.78 ± 1.25C | 13.87 ± 4.57A |
| *Bdellovibrionales* | 0.26 ± 0.08B | | 0.04 ± 0.02C | 0.45 ± 0.14A |
| **Family** |  | |  |  |
| *Anaerolineaceae* | 7.95 ± 5.93B | | 1.78 ± 1.25C | 13.87 ± 4.57A |
| *Bacteriovoracaceae* | 0.15 ± 0.03B | | 0.03 ± 0.02C | 0.39 ± 0.11A |

Different letters (A, B, C) following the abundances indicate a significant difference (P < 0.05) based on Wilcoxon signed rank test.

^a^ Average proportions in 16S cDNA amplicon datasets for the four-depth sediment samples.

^b^ Average proportions in 16S rDNA amplicon datasets for the four-depth sediment samples.

^c^ Average proportions in MT-16S datasets for the four-depth sediment samples.
